# William Warwick James *OBE* FRCS MCh FDS FLS (1874 to 1965)

**DOI:** 10.1177/09677720211064295

**Published:** 2021-12-14

**Authors:** Stanley Gelbier

**Affiliations:** Unit for the History of Dentistry, 4616 King’s College London, London, UK

**Keywords:** Warwick James, maxillofacial surgery, dental elevators, dental, anatomical and palaeontological research

## Abstract

William Warwick James was one of the most inspiring and outstanding dental surgeons of his time, a key researcher in dentistry and zoology and a pioneer in maxillofacial surgery. Most maxillofacial departments hold sets of his dental elevators. He wrote a major wartime work with Benjamin Fickling on the treatment of jaw and facial injuries.

## William Warwick James

Ben Fickling thought that William Warwick James was “the most remarkable dentist of his generation”.^1^ He was born on 20 September 1874 at Wellingborough, Northants, where his self-educated father, also William Warwick James, was a local grocer. He was able to use his father's fine library which allowed him to broaden his knowledge and outlook.^2^ From 1885 to 1891 William attended Wellingborough School, founded in Northamptonshire for boys in 1595.^3^

## Wellingborough School

Amusingly, in its early years there was great rivalry between the school's two masters: the Headmaster teaching Latin, his deputy English, each on separate floors. Eventually the first floor (the Upper School) emerged as the Wellingborough School whilst the Lower School became the town's Grammar School. His younger brother, John Egbert James, was also a pupil at the school, from 1885 to 1893. He was Secretary of the Imperial Chemical Industries (ICI) from 1929 until 1945.

In William's time boys at the school sat Cambridge University Local Examinations. In December 1885 (aged 11) he received a Certificate for under-16s (perhaps the equivalent of GCSE). It does not specify the subjects. It is worth noting that results were shown as Class I, II, III or just Certificate, so William was not in the top tier. In the following December, again a Certificate. He was good at mathematics. Twelve months later he was placed 51st out of the 4981 candidates across the country who sat the Exam Board's Mathematics paper (GCSE equivalent); five Wellingborough Grammar School boys were placed even higher.

In December 1888 (aged 14) William gained a Senior Exams Certificate (for under 18s, so the equivalent of A level). The subject(s) were not mentioned. In the following December he gained another Junior Certificate, and a year later yet another. At the same time he took a further Junior Maths paper, coming 74th out of 5076 candidates.^4^ In December 1891 William gained a Certificate from the College of Preceptors, which represents a high level of attainment in mathematics.

## A life of dentistry

William left the school at the age of 17 years. Having decided to become a dentist he was apprenticed to William Hodgskin Hope, a local practitioner “who found him an eager and apt pupil”.^5^ It would have given him a chance to learn about dental mechanics. This was common at the time and would probably have exempted him from the course at dental school. It would also have allowed him to build up a nest egg to later pay his dental school fees.

In 1894 William went to London to begin his studies, starting with the sciences. At the Dental Hospital of London he qualified with the Licence in Dental Surgery (LDS) in 1898; at the Middlesex Hospital Medical School he gained the MRCS, LRCP in 1902. Within three years he passed the FRCS (Eng) examination.

Warwick James, as he became known, was elected a dental surgeon to the Hospital for Sick Children, Great Ormond Street by the age of 30 years. Three years later he joined the by then ‘Royal’ Dental Hospital (RDH) and in a further six, the Middlesex Hospital. At that time the duties would have been relatively simple, with none of the present high-powered treatments. Although he later resigned from the first two he retained the Middlesex contract until the age of 65. It was there that he did much research at the Bland Sutton Laboratories, often unpaid in the evenings. Like many leading dental surgeons of his era Warwick James also had an extensive private practice, at 2 and 3 Park Crescent, at the north end of Portland Place. One of his patients was Thomas Edward Lawrence (Lawrence of Arabia), who became his friend and left him his music gramophone records. That practice was maintained over his full working life, a very important income-earner as many hospital posts at the time were unpaid honoraries.

## And so to war

When World War I began Warwick James, aged 40, volunteered his services. He first went to France as a civilian to the Anglo-French Unit at the Red Cross Hospital d’Alliances in Yvetot,. There he met Auguste Charles Valadier, a French pioneer of maxillofacial surgery, and Varaztad Kazanjian, an American citizen of Armenian descent, regarded by some as the founder of modern plastic surgery. No doubt he learned much from them, which he carried with him when back in England to work at the 3rd London General Hospital in Wandsworth, south London where he joined William Hern and his colleagues developed highly skilled methods for the repair of extensive facial injuries. Along with surgeon colleague Zachary Cope, he performed many successful mandibular bone-grafts and plastic repairs, all before the introduction of endotracheal intubation and antibiotics.^6^ For many years he maintained contact with patients and arranged annual reunions and dinners.

Warwick James retained links with the Wandsworth hospital after the war. For his amazing work he was awarded a Civilian OBE in 1920. In 1932 he became a member of an important committee on maxillofacial injuries.

## Army Advisory Committee on Maxillo-Facial Injuries

In 1932 the government established an Army Council Advisory Committee on Maxillo-Facial Injuries to report on the management of injuries to the jaws and face in modern warfare. Although he loathed committees he joined Sir Harold Gillies and (later Sir) William Kelsey Fry who had worked together during WWI at the Cambridge Hospital, Aldershot, and latterly at Queen Mary's Hospital, Sidcup.

The committee advised in 1935 that the provision of facilities to treat face and jaw injuries from war should be made at special hospitals rather than in special departments of general hospitals.^7^ Therefore, in the Second World War Emergency Medical Services hospitals were set up which, after the war, continued to operate for plastic and oral and maxillofacial surgery. All but the one at East Grinstead moved to other locations in the next two decades. They became departments where modern plastic surgery was developed in the UK. Several also became the main players in the evolution in oral and maxillofacial surgery.

So Warwick James’ shared recommendations enormously influenced the development of the UK's plastic and oral and maxillofacial surgery.

## An important book and René Le Fort's research

**Figure 1.**
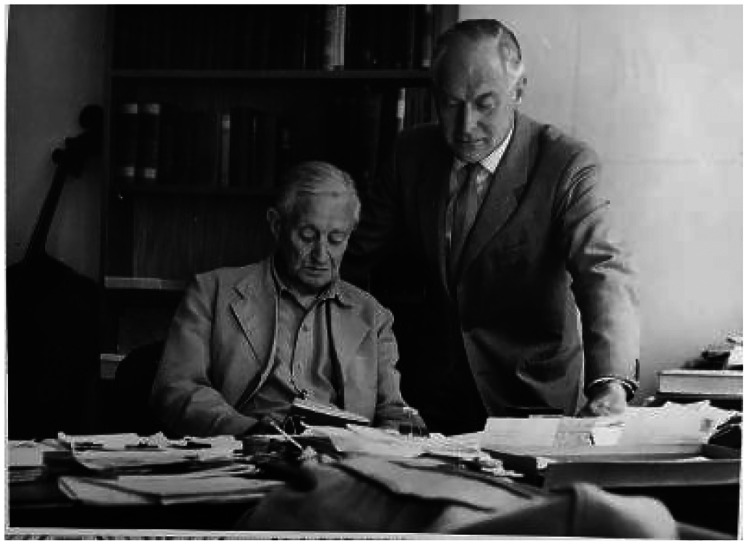
William Warwick James, seated with Ben Fickling.

In the Second World War, because of a lack of information on the treatment of jaw and facial injuries, he wrote a book on the subject in 1940 in collaboration with Ben Fickling (Figure 1).^8^ Much of its source material was provided by his own illicitly-retained notes and records from World War I, which he kept in a basement at home. That was lucky as many official records at Sidcup and Aldershot were destroyed during the war. He and Fickling translated and published^9^ extracts from French surgeon René Le Fort's 1901 research. The latter carried out macabre experiments on 35 cadavers of crash-injured people.^10^ Le Fort inflicted traumas on their faces by dropping cannon balls and striking them with a bat. He then boiled the heads to remove soft tissue and recorded the skeletal results. It was the first time English-speaking surgeons read about it, resulting in a universally accepted classification of mid-facial fractures.

## A lifetime of research

Warwick James developed an early passion for research, conducted at the Dental Hospital, but most of his major work was in the Bland Sutton Laboratories of the Middlesex. He wrote on tooth eruption as early as 1908^11^ and enlarged on the subject in a paper to the British Society for the Study of Orthodontics in 1921.^12^ There is insufficient space in this paper to list and examine all his research papers. However many are covered by Fickling and Boyes.^13^ That enthusiasm stayed with him even beyond retirement. By the age of 86 years he wrote a major book on the teeth and jaws of primates. Many important works appeared in between.

In 1906 the British Dental Association held its annual conference in London. As secretary of its museum committee Warwick James gathered a collection of odontomes.^14^ It was then agreed to have a permanent exhibit. He, Douglas Gabell and Lewin Payne were asked to prepare a book based on his list of specimens. Their 1914 report^15^ presented a better classification of odontomes than the one in existence; that of Sir John Bland, whose dresser Warwick James was for a number of years. The new classification was used for many years.

Warwick James was a careful observer of the clinical scene, for example after tooth extractions.^16^ He was keenly interested in the causes of oral diseases. In 1922 Warwick James collaborated with James Mackintosh and P Lazarus Barlow to research the aetiology of dental caries.^17^ At the beginning of the 20th century there was an increased awareness of the role of micro-organisms in relation to diseases. Although there was a general feeling that pyorrhoea aleveolis (a disease of the tooth's periodontal supporting tissues) was untreatable, important histological research by Warwick James and Edward Arthur Counsell in 1927 was a milestone.^18^

Retirement from practice gave him more time for research. During World War II he worked in London and in the Dental Unit at Birmingham University, the latter with Edgar Manley and Alfred William Wellings. There he made many friends, became an honorary member of the university staff and was awarded an honorary MCh degree in 1944.

As a student he had been present at the Dental Hospital for the last of Sir Charles Tomes’ renowned lectures on dental anatomy. It inspired an interest which developed over time, his researches extended into comparative anatomy and palaeontology. With Welling he researched dental epithelium and its significance on tooth development.^19^ He wrote in 1953 on the succession of teeth in elasmobranchs.^20^ An intensive histological study of tooth dentine in a number of animals was reported in the *Transactions of the Zoological Society of London*.^21^ His important contributions led to several new views on the origin and nature of dentine.

Although by then over 80 years of age, James undertook a major project that was not completed until 1959. He published a volume containing photographic reproductions of the skulls of all the main types of Primates with special regard to the teeth and jaws.^22^ Three photographic views of each specimen were taken and suitable descriptions were added, using skulls at the Natural History Museum, South Kensington. This unique volume was a fitting culmination to a lifetime of research.

Not surprisingly Warwick James was a Fellow of the Zoological and Linnaean Societies and a member of the Geological Association and the Anatomical Society. The Linnean Society of London, founded in 1788, is the world's oldest active society devoted to natural history, named after the Swedish naturalist Carl Linnaeys (1707–1778). His botanical, zoological and library collections have been in its keeping since 1829. Much of his material was obtained from the London Zoo.

**Figure 2.**
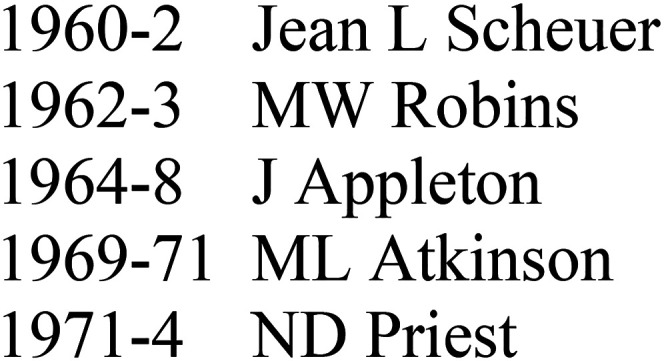
Early recipients of the WWJ scholarship at RDH.

**Figure 3.**
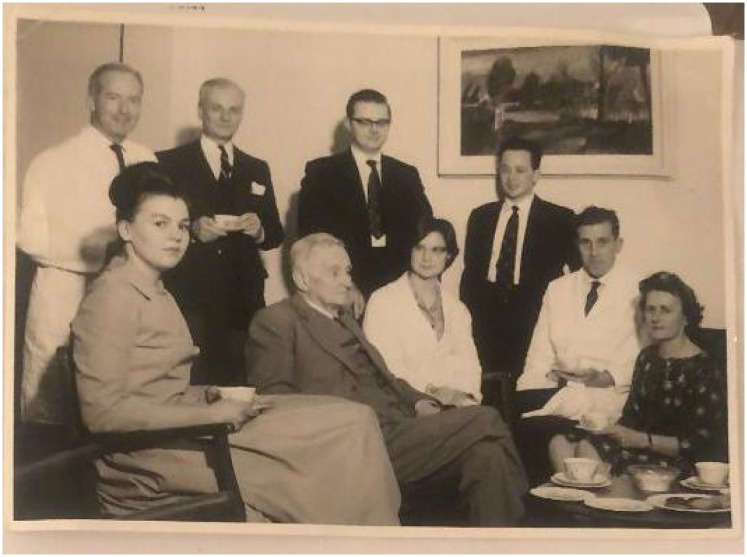
Group in staff room at RDH. Standing left to right are Harry Blackwood (oral pathologist), J McKenzie Briggs (school secretary), Roger Anderson (research assistant) and John Greenspan (research assistant). Seated are Karen Spence (research assistant), William Warwick James, Jean Scheuer (research fellow), Bill Sims (microbiologist) and Beryl Cottell (deputy school secretary).

Warwick James encouraged research by younger dentists. For this purpose he made a generous donation to the Royal Dental in 1960 for a Warwick James Research Fellowship to continue the work of the anatomists who studied the evolution, development, nature and structure of teeth, particularly John and Charles Tomes” in dental anatomy. The early winners are shown in Figure 2. The first recipient was Jean Scheuer who went on to great heights. Figure 3 shows her at RDH with Warwick James and some other colleagues. In 2000 she co-wrote a highly acclaimed *Developmental Juvenile Osteology* for physical anthropologists, archaeologists and forensic pathologist^23^

An eponymous annual Warwick James lectureship was founded at RDH in his honour in 1962 on subjects linked to his ideals.

## Surgical instruments

Warwick James is remembered for his eponymous association with a set of three dental elevators (straight, right and left curved).^24^ William Kelsey Fry popularised their use, for example to aid the removal of roots of fractured teeth. However, despite these instruments being named after him, Warwick James disliked elevators.^25^ This is because they needed a fulcrum which sometimes compressed alveolar bone, which he believed could cause osteitis and post-operative pain.^26^ He preferred to extract teeth with forceps where possible.

## Honours and awards

In 1923 the Royal College of Surgeons of England awarded Warwick James its John Tomes Prize in recognition of his researches. It is given for original scientific work on dental surgery and pathology, dental anatomy and physiology or dental mechanics. When the Faculty of Dental Surgery was founded in that College in 1947 he was one of the earliest dentists to be elected to its Fellowship. In 1945 he had become a life member of the BDA.

## Outside of dentistry

Warwick James and his brother were members of the Alpine Club, a keen mountaineer in his younger days. He enjoyed music and playing the violincello, substantially larger than the violin and viola, with a deeper sound. Warwick James was especially interested in playing chess and solving chess problems.^27^

In 1903 he married Ada Louise Mary (née Froude) of Calne, Wiltshire. Of their six boys and a daughter, two sons became doctors, another a dental surgeon. Ada died in 1948.

**Figure 4.**
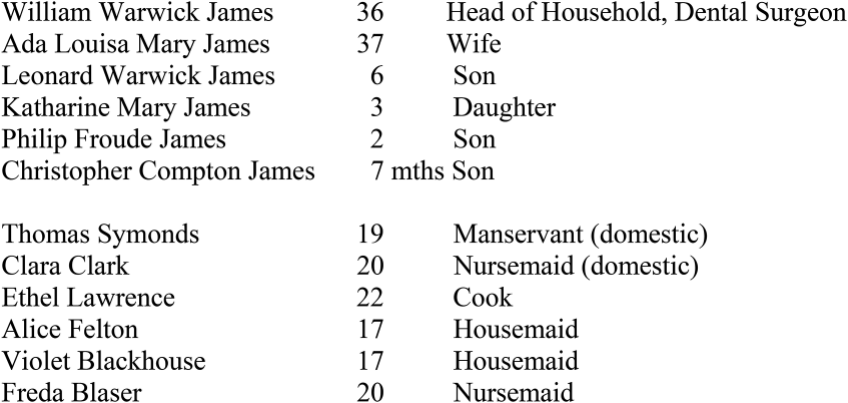
William Warwick James’ household in 1911.

Clearly Warwick James was well off. In the 1911 Census for England and Wales he is shown as living at 2 Park Crescent, Portland Place, west London. The members of his household are listed on Figure 4.

Warwick James died at his home, ‘The Limes’, in Curry Rivel, Somerset on 14 September 1965, just six days before his 91st birthday.
